# Supplementary material for: SIG-DB: Leveraging homomorphic encryption to securely interrogate privately held genomic databases
Source: PLoS Comput Biol. 2018 Sep 4;14(9):e1006454. doi: 10.1371/journal.pcbi.1006454 (PMC6138421; doi:10.1371/journal.pcbi.1006454)
Supplement: S2 Fig — (A) DB held constant at 20,000 bases while query varied from 5,000–20,000 bases. (B) Query held constant at 20,000 bases while DB varied from 5,000–20,000 bases. (DOCX) [file pcbi.1006454.s003.docx]

**Supplemental content:**

**SIG-DB: leveraging homomorphic encryption to Securely Interrogate privately held Genomic DataBases**

Alexander J. Titus^1,2,^*, Audrey Flower^3^, Patrick Hagerty^4^, Paul Gamble^3^, Charlie Lewis^5^, Todd Stavish^3^, Kevin P. O’Connell^1^, Greg Shipley^5^, and Stephanie M. Rogers^1^

^1^B.Next, In-Q-Tel, Arlington, VA, USA

^2^Quantitative Biomedical Sciences, Dartmouth College, Hanover, NH, USA

^3^Lab41, In-Q-Tel, Arlington, VA, USA

^4^CosmiQ, In-Q-Tel, Arlington, VA, USA

^5^Cyber Reboot, In-Q-Tel, Arlington, VA USA

*Corresponding author: [bnext@iqt.org](mailto:bnext@iqt.org)

**Abstract**

Genomic data are becoming increasingly valuable as we develop methods to utilize the information at scale and gain a greater understanding of how genetic information relates to biological function. Advances in synthetic biology and the decreased cost of sequencing are increasing the amount of privately held genomic data. As the quantity and value of private genomic data grows, so does the incentive to acquire and protect such data, which creates a need to store and process these data securely. We present an algorithm for the Secure Interrogation of Genomic DataBases (SIG-DB). The SIG-DB algorithm enables databases of genomic sequences to be searched with an encrypted query sequence without revealing the query sequence to the Database Owner or any of the database sequences to the Querier. SIG-DB is the first application of its kind to take advantage of locality-sensitive hashing and homomorphic encryption to allow generalized sequence-to-sequence comparisons of genomic data.

**Supplemental Figure 2:**


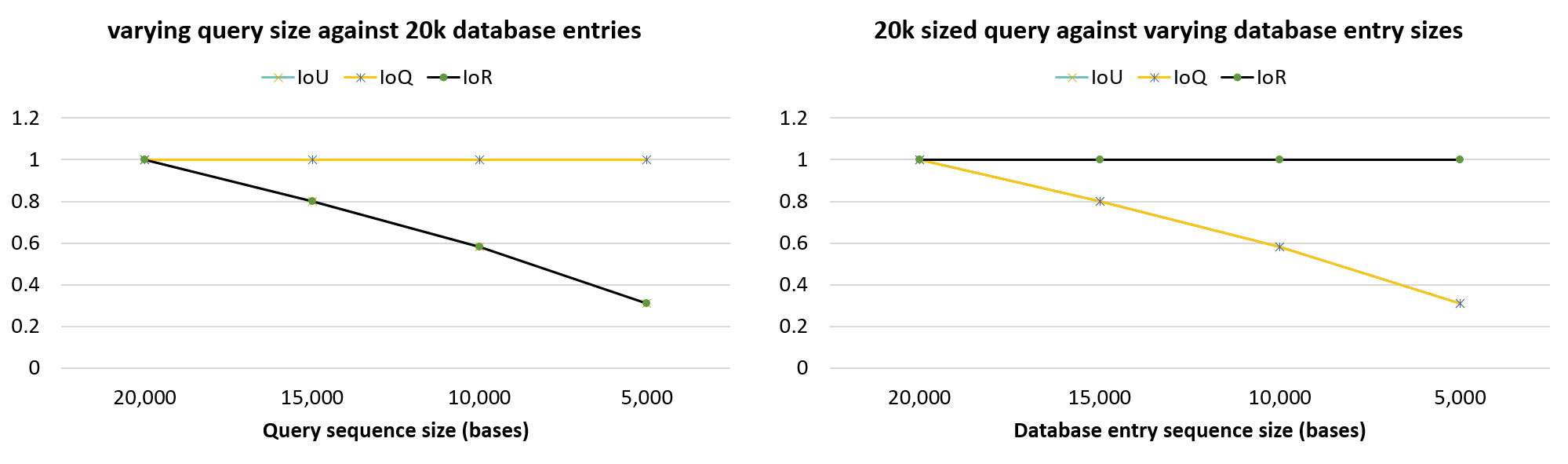


(A)

(B)
