# Supplementary material for: SIG-DB: Leveraging homomorphic encryption to securely interrogate privately held genomic databases
Source: PLoS Comput Biol. 2018 Sep 4;14(9):e1006454. doi: 10.1371/journal.pcbi.1006454 (PMC6138421; doi:10.1371/journal.pcbi.1006454)
Supplement: S4 Fig — Receiver Operator Characteristic (ROC) curves for Paillier-SIG-DB based on each similarity score–(A) IoD, (B) IoU, and (C) IoQ. Query length = 1000 bases and ANI threshold of 0.99. The DB entry length was 1000 bp (top row) with a total of 750,000 query to database entry comparisons and 1,515 of those with ANI scores above the ANI threshold (true positives) and 3000 bp (bottom row) with DB entry length = 3000 bp with a total of 750,000 query to database entry comparisons and 1,708 of those with ANI scores above the ANI threshold. Using IoD as the similarity score when a query is one-third the size of the database entries results in an odd ROC curve representing very poor performance, as shown in (A) bottom row. One possible explanation is that the denominator is larger than the possible intersection space, and as such, we may be washing out the small differences between the intersections of positive and negative examples. In a dataset of only 0.2% positive samples, this leads to broad misclassification. This phenomenon is exactly why we included IoD, IoU, and IoQ as our scoring metrics. (DOCX) [file pcbi.1006454.s005.docx]

**Supplemental content:**

**SIG-DB: leveraging homomorphic encryption to Securely Interrogate privately held Genomic DataBases**

Alexander J. Titus^1,2,^*, Audrey Flower^3^, Patrick Hagerty^4^, Paul Gamble^3^, Charlie Lewis^5^, Todd Stavish^3^, Kevin P. O’Connell^1^, Greg Shipley^5^, and Stephanie M. Rogers^1^

^1^B.Next, In-Q-Tel, Arlington, VA, USA

^2^Quantitative Biomedical Sciences, Dartmouth College, Hanover, NH, USA

^3^Lab41, In-Q-Tel, Arlington, VA, USA

^4^CosmiQ, In-Q-Tel, Arlington, VA, USA

^5^Cyber Reboot, In-Q-Tel, Arlington, VA USA

*Corresponding author: [bnext@iqt.org](mailto:bnext@iqt.org)

**Abstract**

Genomic data are becoming increasingly valuable as we develop methods to utilize the information at scale and gain a greater understanding of how genetic information relates to biological function. Advances in synthetic biology and the decreased cost of sequencing are increasing the amount of privately held genomic data. As the quantity and value of private genomic data grows, so does the incentive to acquire and protect such data, which creates a need to store and process these data securely. We present an algorithm for the Secure Interrogation of Genomic DataBases (SIG-DB). The SIG-DB algorithm enables databases of genomic sequences to be searched with an encrypted query sequence without revealing the query sequence to the Database Owner or any of the database sequences to the Querier. SIG-DB is the first application of its kind to take advantage of locality-sensitive hashing and homomorphic encryption to allow generalized sequence-to-sequence comparisons of genomic data.

**Supplemental Figure 4:**


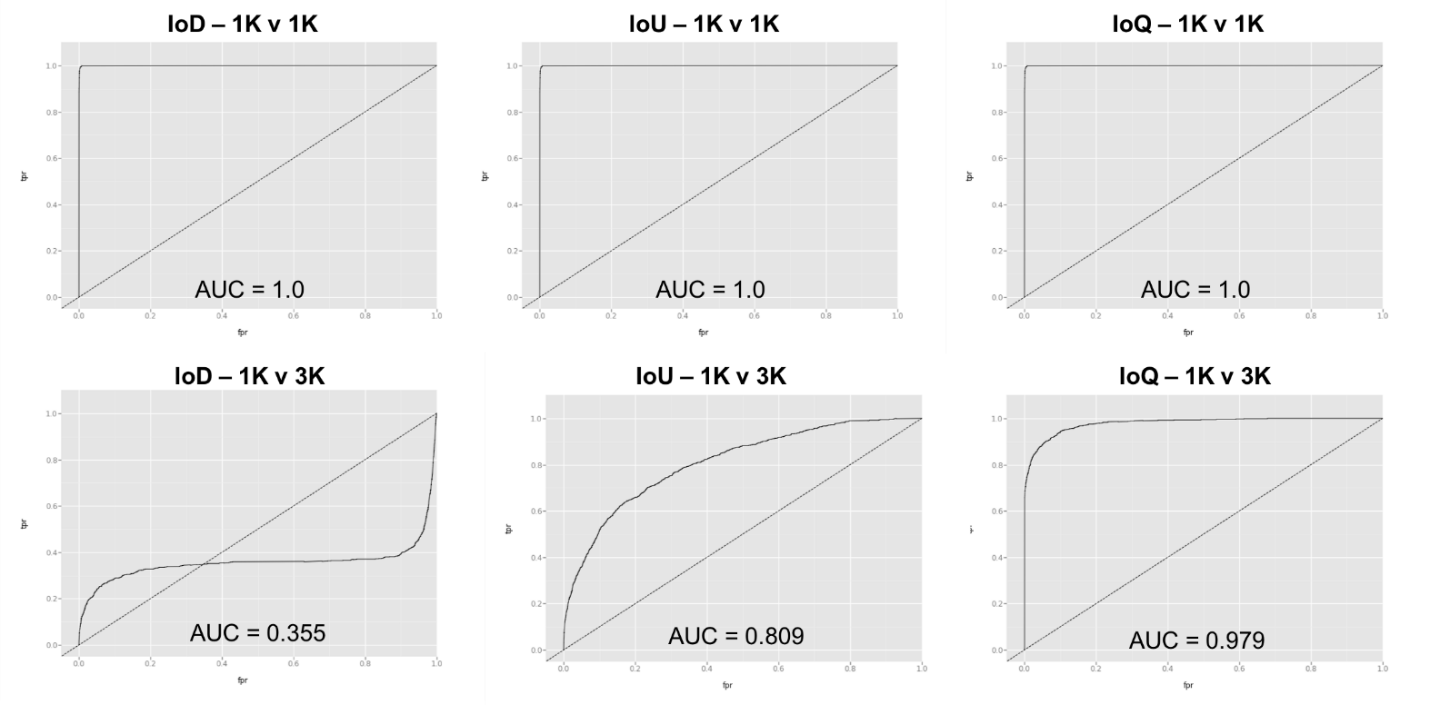


A

B

C
